# Emergence of human West Nile Virus infection in Sri Lanka

**DOI:** 10.1186/s12879-015-1040-7

**Published:** 2015-07-31

**Authors:** Janarthani Lohitharajah, Gathsaurie Neelika Malavige, Anthony Jin Shun Chua, Mah Lee Ng, Carukshi Arambepola, Thashi Chang

**Affiliations:** Faculty of Medicine, University of Colombo, Colombo, Sri Lanka; Department of Microbiology, Faculty of Medical Sciences, University of Sri Jayawardenapura, Sri Jayawardenapura, Sri Lanka; Department of Microbiology, Yong Loo Lin School of Medicine, National University Health System, National University of Singapore, Singapore, Singapore; Department of Community Medicine, Faculty of Medicine, University of Colombo, Colombo, Sri Lanka; Department of Clinical Medicine, Faculty of Medicine, University of Colombo, 25, Kynsey Road, Colombo 08, Sri Lanka

**Keywords:** West Nile virus, Encephalitis, Meningoencephalitis, Sri Lanka

## Abstract

**Background:**

West Nile virus (WNV) has emerged as one of the most common causes of epidemic meningoencephalitis worldwide. Most human infections are asymptomatic. However, neuroinvasive disease characterized by meningitis, encephalitis and/or acute flaccid paralysis is associated with significant morbidity and mortality. Although outbreaks have been reported in Asia, human WNV infection has not been previously reported in Sri Lanka.

**Methods:**

Sera and cerebrospinal fluid (CSF) from 108 consecutive patients with a clinical diagnosis of encephalitis admitted to two tertiary care hospitals in Colombo, Sri Lanka were screened for WNV IgM antibody using enzyme-linked immunosorbent assay. Positive results were confirmed using plaque reduction neutralization test (PRNT). Patient data were obtained from medical records and by interviewing patients and care-givers.

**Results:**

Three of the 108 patients had WNV IgM antibody in serum and one had antibody in the CSF. The presence of WNV neutralizing antibodies was confirmed in two of the three patients using PRNT. Two patients had presented with the clinical syndrome of meningoencephalitis while one had presented with encephalitis. One patient had CSF lymphocytic pleocytosis, one had neutrophilic pleocytosis while CSF cell counts were normal in one. CSF protein showed marginal increase in two patients.

**Conclusions:**

This is the first report of human WNV infection identified in patients presenting with encephalitis or meningoencephalitis in Sri Lanka. There were no clinical, routine laboratory or radiological features that were distinguishable from other infectious causes of meningoencephalitis.

## Background

West Nile virus (WNV) is one of the most widely distributed, medically important arboviral infections that has caused major outbreaks in many parts of the world [1]. Although the initial WNV outbreaks were confined to rural areas in Africa [1], it is now the leading cause of encephalitis in USA, Europe and Australia [2, 3]. Initial WNV outbreaks were associated with only a few cases of severe neurological diseases. However, neuroinvasive disease is now more frequent and the case fatality rates range from 4.2 to 18.6 % in more recent epidemics [1, 4, 5]. WNV is considered to be one of the most important emerging flaviviral infections in the world, due to the increase in the number of cases with expansion in geographical distribution, and its association with severe neurological disease [6, 7].

WNV is a zoonotic infection, where the virus cycles between mosquitoes and birds. The *Culex* genus of mosquitoes are the main vectors while passerine birds act as amplifying hosts [1]. Humans and mammals are usually incidental, dead-end hosts as viral titers in mammals are insufficient to infect mosquitoes for further transmission to other mammals [8]. WNV results in neuroinvasive disease in less than 1 % (approximately 1 in 150) of infected individuals, while asymptomatic infections occur in around 80 % [9, 10]. Approximately 20 % of infected individuals develop WN fever, which is an undifferentiated flu-like illness that occurs 2–14 days after an infectious mosquito bite. This is characterized by fever, myalgia, gastrointestinal symptoms and sometimes a macular-papular rash [9]. WN fever may mimic the clinical syndromes of other flavivirus infections such as dengue fever. WNV neuroinvasive disease manifests as meningitis, encephalitis, asymmetric acute flaccid paralysis or a mixed pattern of these syndromes. Encephalitis is more common than meningitis in older age groups, and is commonly associated with extrapyramidal features while acute flaccid paralysis may lead to respiratory paralysis. After the acute infection, many patients experience persistent symptoms, such as fatigue, memory impairment, weakness, headache, and balance problems.

**Fig. 1:**
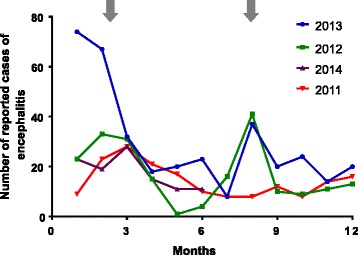
Temporal distribution of notified cases of encephalitis in Sri Lanka. *Arrows* indicate peak periods of highest frequencies reported during the year

Encephalitis is a notifiable disease in Sri Lanka and annually, 165–220 cases are reported to the Epidemiology Unit of Sri Lanka. However, in 2013, a large outbreak of encephalitis occurred with 141 cases of encephalitis being reported in the first 2 months of the year. Although cases of encephalitis occur throughout the year in Sri Lanka, there are usually two peaks in the number of cases reported (Fig. 1) [11–13]. These peaks coincide with the monsoon rain seasons in Sri Lanka and are likely to reflect an increase in vector densities caused by increased mosquito breeding in stagnant collections of rainwater. These two peaks also coincide with the latter part of the migratory bird season. This provides the requisite environment for the maintenance of the zoonotic WNV life-cycle between mosquitoes and birds. The *Culex* genus of mosquitoes are endemic while passerine birds are both endemic and migratory in Sri Lanka. Although human WNV infection has not been previously reported in Sri Lanka, it has reportedly caused several outbreaks in neighboring India, including Kerala and Tamil Nadu, which are in close proximity to Sri Lanka [14, 15].

Given the conducive environment for transmission, WNV has the potential to emerge as a major cause of meningoencephalitis in Sri Lanka. In this study, we report the first identification of human WNV infection in Sri Lanka in patients presenting with meningoencephalitis.

## Methods

### Patients

108 patients with clinical syndromes of encephalitis or meningoencephalitis, who were admitted to two of the largest tertiary care hospitals in Sri Lanka (the National Hospital of Sri Lanka and the Lady Ridgeway Hospital for Children), were included in the study following informed written consent. In the instances where children were recruited, informed written consent was obtained from the guardian. The study was approved by the Ethic Review Committees of the Faculty of Medicine, University of Colombo and those of the two hospitals. Clinical and laboratory data including cerebrospinal fluid (CSF) analysis, CT or MRI scan results were recorded. Serum and CSF were obtained from all patients and stored at -80 °C until analyzed.

### ELISA for detection of WNV

WNV Detect™ IgM enzyme-linked immunosorbent assay (ELISA) (InBios, USA) was used for the initial screening for WNV infection. In sera and CSF that tested positive for WNV, ELISA for Japanese encephalitis virus (JEV) and dengue virus were also performed to eliminate potential false positive results due to the cross-reactivity between the antibodies of these flaviviruses. JE Detect™ IgM ELISA (InBios, USA) was used for the detection of antibodies in human serum to determine exposure to JEV. Calculation of the immune status ratio (ISR) was done according to the manufacturer’s instructions that consider ISR units of > 6.0 as positive for acute JEV infection. Serum and CSF were also tested for the presence of anti-dengue virus antibodies by using a commercial capture-IgM and IgG ELISA (Panbio, Brisbane, Australia). Panbio units of > 11 were considered positive for acute dengue infection.

### Plaque reduction neutralization test (PRNT) for WNV

PRNT was done at the National West Nile virus reference laboratory of the National University of Singapore. Serum samples were inactivated at 56 °C for 30 min, and diluted 100 times in RPMI medium containing 2 % FBS (virus diluent). A 10-step 2-fold serial dilution was carried out using virus diluent before 500 PFU of WNV was added to 500 μl of each serum dilution. The rest of the assay was performed as previously described [16].

### WNV specific RT-PCR

Viral RNA was extracted from serum using QIAmp viral RNA mini kit (Qiagen, Germany). RNA was reverse transcribed and the PCR was performed by using primer and conditions as previously described [17].

### Laboratory criteria for diagnosis of WNV infection

WNV infection was diagnosed as ‘definite’ in patients with WNV-specific IgM in serum or CSF (detected using ELISA) and confirmed with the WNV-specific PRNT assay. WNV infection was diagnosed as ‘probable’ in patients with WNV-specific IgM in serum or CSF, but without JEV or dengue virus IgM (detected using ELISA) in the absence of WNV-specific PRNT confirmation.

## Results

Of the 108 patients recruited into the study, 3 were diagnosed to have WNV neuroinvasive disease based on their clinical syndromes and confirmed by laboratory results (Table 1). WNV IgM antibodies were detected in the serum of all three patients and the CSF of Patient #2. CSF of the other two patients could not be tested due to insufficient quantities of CSF. Because antibodies to JEV and dengue virus can cross-react and give rise to false-positive results in the WNV IgM ELISA test [18, 19], we tested both sera and CSF for the presence of JEV-specific IgM antibodies and dengue virus-specific IgM and IgG antibodies. Cross-reactivity was evident in Patients #1 and #2 (Table 1). None of the samples tested positive for WNV on RT-PCR. In order to confirm WNV infection, the seropositive sera of Patients #1 and #2 were analysed for the presence of neutralizing antibodies to WNV, using the PRNT assay. Both patients had PRNT50 neutralizing antibody titers of 800 against WNV. Accordingly, Patients #1 and #2 were diagnosed to have definite WNV infection while Patient #3 was diagnosed to have probable WNV infection.

**Table 1:** Results of IgM-ELISA of West Nile, Japanese encephalitis and dengue virus in serum and CSF of the reported patients

	Patient #1	Patient #2	Patient #3
WNV Serum	35.343	23.433	7.032
WNV CSF	ND	37.518	ND
JEV Serum	12.656	31.100	1.308
JEV CSF	1.746	29.344	ND
Dengue Serum	49.051	4.642	8.825
Dengue CSF	8.204	11.211	ND
PRNT value for WNV			

Cut off values for WNV antibodies: positive >5.66; equivocal 4.47–5.66; negative <4.47

Cut off values for JEV antibodies: positive >6.00; equivocal 4.00–6.00; negative <4

Cut off values for Dengue antibodies: positive >11; equivocal 9–11; negative <9

*CSF* cerebrospinal fluid, *JEV* Japanese encephalitis virus, *ND* not done, *WNV* West Nile virus

The clinical and laboratory features of these patients are shown in Table 2. Two were males and one female, with ages ranging from 17 to 49 years. All three patients presented with abrupt onset fever and altered sensorium, predominantly confusion. Patients #2 and #3 had prominent headache, and Patients #1 and #3 developed generalised tonic-clonic seizures. Neck rigidity and increased tone was noted in Patients #2 and #3. Neutrophil leucocytosis was noted in the peripheral blood of two patients. Lymphocytic pleocytosis was noted in the CSF of one patient while neutrophils predominated in the CSF of Patient #2. CSF analysis was normal in Patient #1. CSF protein was marginally elevated in two of the three patients. Generalised cerebral oedema was noted in Patients #2 and #3 on CT brain imaging. MRI brain was done in only Patient #3 which showed meningeal enhancement. All three patients were treated with empirical intravenous antibiotics and aciclovir for variable periods and all patients recovered to their pre-morbid states.

**Table 2:** Clinical and laboratory features of the reported patients

	Patient #1	Patient #2	Patient #3
Age (years) and sex	17, male	36, female	49, male
Clinical presentation			
Fever	+ (high grade, 3 days)	+ (high grade, 3 days)	+ (high grade, 5 days)
Headache	-	+ (severe, 3 days)	+ (5 days)
Nausea/vomiting	-	+	+
Altered level of consciousness	+ (confusion)	+ (confusion)	+ (agitation, 5 days)
Seizures	2 x GTCS	-	2 x GTCS
Other	Asthenia, polyarthralgia	Photophobia	-
Past medical history	Nil	Nil	Hypertension
Neurological examination	GCS 14/15; confused; no focal signs, neck stiffness or paralysis; normal cranial nerves and tendon reflexes.	GCS 11/15; neck stiffness and increased limb tone; normal tendon reflexes; no focal signs; disconjugate gaze but otherwise normal cranial nerves.	GCS 10/15; neck stiffness; increased limb tone and tendon reflexes.
General and other systems examination	Normal; no rash; BP 110/70 mmHg	Normal; no rash; BP 100/60 mmHg	No rash; BP 140/90 mmHg
Blood investigations			
Haemoglobin (g/dl)	14.8	10.5	14.3
White cell count (x 10^9^/L)	5,400	12,200 N85%	13,200
Platelet count (x 10^9^/L)	154,000	235,000	263,000
ESR (mm/h)	Not done	Not done	03
CRP (mg/L)	ND	96	ND
ALT (U/L)	46	ND	106
AST (U/L)	85	ND	156
SAP (U/L)	221	ND	157
Creatinine (μmol/L)	60	ND	66
Sodium (mmol/l)	140	ND	118
Potassium (mmol/l)	3.9	ND	3.9
Cerebrospinal fluid			
Colour	Colourless	Colourless	Colourless
Protein (mg/dl)	42	48	26
Glucose	4.3	2.2	2.7
Lymphocytes	01	00	10
Polymorphs	00	64	02
Erythrocytes	01	32	3500
Gram stain	Negative	Negative	Negative
Culture	Negative	Negative	Negative
Other			PCR TB: negative
Random plasma glucose (mmol/l)	6.5	4.5	8.1
Neuroimaging			
CT brain	Normal	Cerebral oedema	Cerebral oedema
MRI brain	Not done	Not done	Meningeal enhancement
EEG	Bilateral slow wave discharges	Bilateral slow wave discharges	Bilateral slow wave discharges
Treatment	IV aciclovir, dexamethasone and cefotaxime; stopped after 4 days. Oral phenytoin 100 mg BD continued.	IV ceftriaxone, aciclovir and dexamethasone; latter 2 stopped after 2 days.	IV ceftriaxone, aciclovir and thiamine; IV antibiotics continued for 12 days. Thiamine stopped after 8 days.
Outcome	Complete recovery	Complete recovery	Complete recovery

Clinical findings and laboratory investigations shown are at the time of presentation

*ALT* alanine transaminase, *AST* aspartate transaminase, *BP* blood pressure, *CRP* c-reactive protein, *CT* computerized tomography, *EEG* electroencephalography, *ESR* erythrocyte sedimentation rate, *GCS* Glasgow coma scale score, *GTCS* generalized tonic-clonic seizure, *IV* intravenous, *MRI* magnetic resonance imaging, *ND* not done, *PCR* polymerase chain reaction, *SAP* serum alkaline phosphatase, *TB* tuberculosis

## Discussion

This is the first report of human WNV infection in Sri Lanka presenting with encephalitis and meningoencephalitis. Three of 108 patients (2.8 %) presenting with a clinical diagnosis of encephalitis or meningoencephalitis admitted to hospital were diagnosed to have WNV infection retrospectively when serum and CSF were screened for WNV IgM antibodies. Since dengue and Japanese encephalitis are endemic in Sri Lanka and anti-flavivirus antibodies may cross-react in ELISA to give false-positive results, WNV infection in Patients #1 and #2 were confirmed by specific PRNT assay.

Detection of WNV-specific IgM antibodies in serum and CSF remains the gold standard for the diagnosis of human WNV disease. IgM antibodies to WNV are usually detectable by ELISA in 75 % by day 4 post-infection and 95 % by day 7 post-infection in WNV infected patients [20]. Since IgM antibodies do not readily cross the blood brain barrier, detection of WNV IgM in CSF is diagnostic of neuroinvasive disease. WNV IgM antibody was detected in the CSF in Patient #2. However, unlike most IgM responses, WNV IgM antibody can persist for 6 months or longer in both serum and CSF [21]. The PRNT is the most specific diagnostic test for WNV and can help distinguish serologic cross-reactions caused by other flaviviruses. However, this test is not commercially available, is tedious, and can only be performed in laboratories with the relevant capabilities. Nucleic acid amplification for viral detection is highly specific, but is of limited sensitivity (less than 15 % for serum) since viraemia occurs early before the onset of symptoms, is of low titer and is short-lived [22].

Peripheral blood counts can be normal in patients with WNV neuroinvasive disease, or can demonstrate anaemia, thrombocytopenia, leukocytosis or leukopenia [20]. Typical CSF findings in WNV neuroinvasive disease include moderate lymphocytic pleocytosis (usually <500 cells/μl) with elevated protein (usually <150 mg/dl) and slightly low or normal levels of glucose [23]. However, neutrophils may predominate in early infection in up to 45 % of patients, while 3–5 % of patients with meningitis or encephalitis may have normal CSF cell counts [23]. The presence of abnormalities on neuroimaging has been variable ranging from none to 70 % of reported cases [20].

Of the three patients reported here, two (Patient #2 and #3) presented with clinical features of meningoencephalitis while one presented with encephalitis indistinguishable from other infectious aetiologies presenting with similar syndromes. Although the association of extrapyramidal features and flaccid paralysis is reported to be common in WNV neuroinvasive disease [20], our patients did not demonstrate such distinguishing clinical characteristics. Of the two patients who had peripheral neutrophil leucocytosis, in Patient #3, it is likely to be related to seizures given that the inflammatory marker (ESR) was normal. Bacteriological screening of all three patients, which included blood culture and CSF Gram stain and culture, were negative. The presence of neutrophil predominance in CSF has been suggested as a possible diagnostic clue of WNV infection [23], but only Patient #2 of our three patients had CSF neutrophilic pleocytosis.

## Conclusion

In summary, the clinical, routine laboratory or radiological features of our patients did not provide any diagnostic clues of WNV infection; diagnosis was established based on serological testing for WNV and excluding other possible aetiologies. It is thus reasonable that all patients presenting with a clinical syndrome of meningitis, encephalitis, asymmetric acute flaccid paralysis or any combination of these three syndromes be screened for WNV IgM since this report has now established the occurrence of human WNV disease in Sri Lanka. An accurate diagnosis will help avoid inappropriate treatment and allow correct prognostication. Moreover, this will enable timely evaluation of possible outbreaks of a vector-borne disease that has the potential to emerge as a major cause of meningoencephalitis as it has done in other WNV endemic countries such as the United States of America. This will also allow the Sri Lankan public health authorities to institute timely preventive measures. By extrapolating that neuroinvasive disease occurs in 1 in 150 to 1 in 250 WNV infections [24], our report of three cases would imply that there would be between 450 and 750 cases of WNV infection. As our sample included only patients admitted to two major hospitals in Colombo, Sri Lanka, and since it did not include patients presenting with acute flaccid paralysis, the actual number of WNV infections is likely to be much higher than our estimate.

## Abbreviations

WNV: West Nile virus
CSF: Cerebrospinal fluid
JEV: Japanese Encephalitis Virus
MRI: Magnetic Resonance Imaging
FBS: Fetal bovine serum
PRNT: Plaque reduction neutralization test
